# Hydrolytic Exoenzymes Produced by Bacteria Isolated and Identified From the Gastrointestinal Tract of Bombay Duck

**DOI:** 10.3389/fmicb.2020.02097

**Published:** 2020-08-26

**Authors:** Tanim J. Hossain, Sumaiya I. Chowdhury, Halima A. Mozumder, Mohammad N. A. Chowdhury, Ferdausi Ali, Nabila Rahman, Sujan Dey

**Affiliations:** ^1^Department of Biochemistry and Molecular Biology, University of Chittagong, Chattogram, Bangladesh; ^2^Department of Microbiology, University of Chittagong, Chattogram, Bangladesh; ^3^Department of Biology, Chittagong Sunshine College, Chattogram, Bangladesh

**Keywords:** Bombay duck, gut bacteria, extracellular hydrolytic enzyme, exoenzyme, protease, lipase, pectinase, 16S rRNA gene sequence

## Abstract

Bacteria producing hydrolytic exoenzymes are of great importance considering their contribution to the host metabolism as well as for their various applications in industrial bioprocesses. In this work hydrolytic capacity of bacteria isolated from the gastrointestinal tract of Bombay duck (*Harpadon nehereus*) was analyzed and the enzyme-producing bacteria were genetically characterized. A total of twenty gut-associated bacteria, classified into seventeen different species, were isolated and screened for the production of protease, lipase, pectinase, cellulase and amylase enzymes. It was found that thirteen of the isolates could produce at least one of these hydrolytic enzymes among which protease was the most common enzyme detected in ten isolates; lipase in nine, pectinase in four, and cellulase and amylase in one isolate each. This enzymatic array strongly correlated to the previously reported eating behavior of Bombay duck. 16S rRNA gene sequence-based taxonomic classification of the enzyme-producing isolates revealed that the thirteen isolates were grouped into three different classes of bacteria consisting of eight different genera. *Staphylococcus*, representing ∼46% of the isolates, was the most dominant genus. Measurement of enzyme-production via agar diffusion technique revealed that one of the isolates which belonged to the genus *Exiguobacterium*, secreted the highest amount of lipolytic and pectinolytic enzymes, whereas a *Staphylococcus* species produced highest proteolytic activity. The *Exiguobacterium* sp. expressing a maximum of four hydrolases, appeared to be the most promising isolate of all.

## Introduction

The gastrointestinal (GI) tract of fishes, like those of other vertebrate-hosts, harbors a complex community of bacteria that significantly influence host’s physiology via metabolic interactions (Sommer and Bäckhed, 2013; Romero et al., 2014; Krishnan et al., 2015; Wang et al., 2018). Producing a diverse array of extracellular hydrolytic enzymes, the intestinal flora carry out a wide range of catabolic and biotransformation reactions which are distinct from, but complement the metabolic processes of the host (Krishnan et al., 2015; Banerjee and Ray, 2017; Egerton et al., 2018; Rowland et al., 2018). Several papers have already reported the purification and characterization of a number of hydrolytic exoenzymes such as protease, lipase, amylase, cellulase and chitinase, among others, from the GI bacteria of various freshwater and marine fishes (Ray et al., 2012). Some of these enzymes were found largely diversified in their enzymatic properties and substrate specificities (Sugita et al., 1996; Bairagi et al., 2002; Saha et al., 2006; Ray et al., 2007, 2012; Esakkiraj et al., 2008; Lazado et al., 2012; Ananthi et al., 2014; Kawashima et al., 2016). Fish GI bacteria therefore represent a dynamic source of hydrolytic enzymes for potential applications in many industries such as biotechnology, food, pharmaceuticals, aquaculture, detergent, paper, textiles etc. The GI bacteria of marine fishes are of particular interest in this regard because microbial enzymes from marine environment are renowned for their extraordinary chemical and metabolic abilities (Debashish et al., 2005; Sarkar et al., 2010; Zhang and Kim, 2010). Being the largest ecosystem on earth, the marine environment constitutes one of the richest reserves of bacteria, majority of which remain entirely unexplored and unexploited (Das et al., 2006; Penesyan et al., 2010; Finore et al., 2014). This massive bacterial population is considered as a potent source of novel enzymes and many other bioactive compounds (Newman and Hill, 2006; Lee et al., 2010; Rao et al., 2017; Romano et al., 2017; Sivaperumal et al., 2017). Besides, compared to the enzymes of terrestrial origin, the marine-derived bacterial enzymes, through millions years of evolution, acquired additional characteristics that help the bacteria adapt to the extreme and constantly changing marine environments (de Carvalho and Fernandes, 2010; Zhang and Kim, 2010; Dash et al., 2013; Nikolaivits et al., 2017). With large catalytic activity and flexibility, the marine bacterial enzymes can work in conditions like high pressure, high salinity, high viscosity and high and low temperatures (Zhang and Kim, 2010; Trincone, 2011; Nikolaivits et al., 2017). Some of the enzymes acquire unique chemical or stereochemical properties such as substrate specificity and enantioselectivity, which can be exploited in the chemical and pharmaceutical industries (Trincone, 2010; Parte et al., 2017). Since GI tract of fishes is an open system constantly interacting with the surrounding environment, the diverse bacterial population of the marine environment, taking advantage of their uninterrupted access to the GI tract, forms a major portion of the GI microbiota of fishes (Mondal et al., 2010). Moreover, in comparison with the neighboring environment, the fish GI tract is relatively richer in nutrients and therefore offers a favorable growth environment for the bacteria (Mondal et al., 2010). Hence, studying GI bacteria of marine fishes is of great importance for the discovery of beneficial strains as a promising source of novel biocatalysts.

This work attempts to isolate and characterize extracellular hydrolase producing GI bacteria of Bombay duck (*Harpadon nehereus*), a brackish-water marine lizardfish which has a wide and discontinuous distribution along the coasts of China, India, Bangladesh, East Africa, Malaya and Indonesia (Bapat, 1967; Behera et al., 2015; Zhang et al., 2017). This fish is voracious, carnivorous and cannibalistic in its eating behavior (Kurian, 2000; Balli et al., 2006; Ghosh, 2014; He et al., 2018) with its diet consisted mainly of prawn, shrimp, fish, fish larva and crab (Khan et al., 1992; Balli et al., 2006; Ghosh, 2014; Zhang and Jin, 2014). Presence of plant matters has also been reported in a few studies, though in very small amounts (Mookerjee et al., 1946; Pillay, 1953; Bapat, 1970). It is known that eating habit or, the type of diet is a key factor shaping the composition of gut microbiota (Bairagi et al., 2002, p. 1; Ray et al., 2012; Senghor et al., 2018). Prevalence of protein and fat in the diet of Bombay duck, and lack of plant-derived polysaccharides such as cellulose, starch, chitin, pectin, xylan etc. indicate that the bacteria having extracellular proteolytic and lipolytic activity might predominate in its gut microbiota in comparison to those having cellulolytic, amylolytic, chitinolytic, pectinolytic, or, xylanolytic activity. Each of these hydrolytic enzymes has numerous applications in many industrial bioprocesses and therefore is of great commercial value (Uhlig, 1998; Kirk et al., 2002; Sarrouh et al., 2012; Gurung et al., 2013; Liu and Kokare, 2017). Despite the biotechnological prospects as well as its importance to the host, fundamental knowledge on the gut-associated microbiota of Bombay duck and its hydrolytic enzyme potential has still remained unexplored.

In the present study, GI bacteria of Bombay duck (hereafter referred to as BDGB) were isolated with the aim of evaluating their hydrolytic capacities for potential applications in aquaculture and biotechnology. The isolated strains were examined for the production of five different hydrolytic enzymes such as protease, lipase, pectinase, cellulase and amylase, and the enzyme-producing strains were characterized based on their 16S rRNA gene sequences. This is the first report about the isolation and genetic identification of the enzyme producing gut bacteria of Bombay duck.

## Materials and Methods

### Preparation of Intestinal Sample

For isolation of the GI bacteria, intestinal sample was prepared separately from two Bombay duck fishes that were purchased from a seaside fish market of Chattogram city. The two fishes were 19.7 and 20.02 cm in length and 85.7 and 87 g in weight, respectively. Entire GI tract of each fish was aseptically dissected and washed thoroughly with sterile distilled water (Bairagi et al., 2002). The intestinal contents were then squeezed out and inside of the intestine was rinsed well with water, and both were mixed together to obtain the intestinal sample.

### Isolation of Bacteria and Culture Conditions

Five-fold serial dilutions of the intestinal sample were prepared in sterile distilled water and 100-μL aliquot of each dilution was spread on the surface of nutrient agar (NA; 5 g/L peptone, 3 g/L yeast extract, 5 g/L NaCl, 18 g/L agar; pH 7) and Luria-Bertani (LB) agar (10 g/L tryptone, 5 g/L yeast extract, 10 g/L NaCl, 18 g/L agar; pH 7). After incubation at 30°C for 24 to 48 h, colonies with distinct morphological appearances were selected, restreaked and subcultured on fresh media until pure cultures were obtained (Hossain et al., 2011). The cultures were preserved at −40°C as 20% v/v glycerol stocks in LB or nutrient broth. For all analyses described below, cultures were first activated by inoculating cells from the glycerol stocks in nutrient broth or LB media and after incubation overnight at 30°C, OD_600_ of the overnight cultures was adjusted to 0.8.

### Screening Isolates for Extracellular Hydrolytic Enzyme Production

The isolated bacteria were screened for the production of protease, lipase, pectinase, cellulase and amylase enzymes in agar plate assay. Activated culture of each isolate was streaked on agar media containing suitable substrate specific for each of the enzyme activities. For example, gelatin, Tween-80, pectin, carboxymethylcellulose (CMC) and starch were used for the detection of proteolytic, lipolytic, pectinolytic, cellulolytic and amylolytic activities, respectively. After incubation at 30°C for 48 h, the culture-media were treated with specific staining solutions as described below. Formation of zones of clear halo surrounding the colonies indicated presence of the respective enzymes. For the detection of proteolytic activity, the isolates were inoculated onto gelatin-agar media (10 g/L gelatin, 5 g/L tryptone, 1 g/L glucose, 2.5 g/L yeast extract, 20 g/L agar; pH 7) and incubated at 30°C for 48 h followed by staining the media with mercuric chloride solution (150 g/L HgCl_2_ in 20% v/v HCl). Development of transparent circles around the colonies indicated a positive reaction (Fry et al., 1994). Similarly for lipolytic activity, the isolates were inoculated on Tween 80-agar media (15 mL/L Tween 80, 5 g/L tryptone, 2.5 g/L yeast extract, 5 g/L NaCl, 20 g/L agar; pH 7) and incubated at 30°C for 48 h. The appearance of clear halos after staining with methyl red solution (0.2 g/L methyl red in 95% ethanol) indicated the presence of lipolytic activity (Samad et al., 1989). For pectinolytic activity, isolates grown on pectin-agar media (5 g/L pectin, 5 g/L tryptone, 2.5 g/L yeast extract, 5 g/L NaCl, 15 g/L agar; pH 7) were flooded with potassium iodide solution (20 g/L potassium iodide and 10 g/L iodine) and examined for the appearance of clear zones to confirm pectinase production (Soares et al., 1999). For the determination of cellulolytic activity, the isolates were inoculated onto CMC-agar plates (10 g/L CMC, 2 g/L tryptone, 4 g/L KH_2_PO4, 4 g/L Na_2_HPO_4_, 0.2 g/L MgSO_4_.7H_2_O, 0.001 g/L CaCl_2_, 0.001 g/L FeSO_4_.7H_2_O, 20 g/L agar; pH 7). After incubation at 30°C for 48 h, the plates were first stained with Congo red solution (2 g/L) for 10 min and then destained with 1 M NaCl for 15 min; halo zones surrounding the colonies indicated cellulase production (Meddeb-Mouelhi et al., 2014). For amylolytic activity, bacteria grown on starch-agar media (10 g/L soluble starch, 5 g/L tryptone, 3 g/L yeast extract, 20 g/L agar; pH 7) were flooded with potassium iodide solution; transparent zones surrounding the colonies indicated amylase production (Amoozegar et al., 2003).

### Measurement of Enzyme-Production

Amount of enzyme-production by the isolates was determined by agar diffusion method. The isolates were grown on media containing specific substrates for the respective enzymes as performed in the screening experiment described above and diameter of the zones of clearance and that of the colonies were measured. Amount of the enzyme produced was then calculated, and expressed as enzyme intensity (EI) where EI = (colony diameter + halo zone diameter)/colony diameter (Aleem et al., 2018; Ashok et al., 2019). Each experiment was performed in triplicate and averaged.

### 16S rRNA Gene Amplification and Sequencing

To amplify 16S rRNA genes of the enzyme-producing isolates, their genomic DNA was extracted using a Maxwell 16 Blood DNA Purifcaton Kit (Promega, Madison, WI, United States) according to the manufacturer’s recommendations. Nearly full length of the 16S rRNA gene was amplified from the genomic DNA by PCR using the universal primers 27F (5′-AGAGTTTGATCCTGGCTCAG-3′) and 1492R (5′-GGTTACCTTGTTACGACTT-3′) (Miyoshi et al., 2005) in a 20-μL reaction volume which contained 10 μL GoTaq G2 Hot Start Master Mix (Promega), 1 μL genomic DNA (25 to 65 ng/μL), 1 μL of each primer (10 to 20 pM) and water. The PCR program consisted of an initial denaturation step at 95°C for 3 min followed by 35 rounds of temperature cycling comprising denaturation at 95°C for 30 s, annealing at 48°C for 30 s, and extension at 72°C for 90 s, followed by a final extension at 72°C for 5 min and cooling to 4°C. The PCR products were purified using a Wizard SV Gel and PCR Clean-Up System (Promega) and subjected to sequencing with the same primers that were used for the original PCR amplification using a BigDye Terminator v3.1 Cycle Sequencing Kit (Applied Biosystems) according to the manufacturer’s instructions. The sequences have been deposited in the National Center for Biotechnology Information (NCBI) Data Bank under accession numbers (ACNs) MN611242 to MN611254.

### Analysis of 16S rRNA Gene Sequences

Sequence similarity of the 16S rRNA genes with those in the database were performed by NCBI blastn suite (Johnson et al., 2008), EzBioCloud’s 16S-based ID (database version 2019.08.06) (Yoon et al., 2017) and RDP seqmatch (Cole et al., 2014). The top-hit database strain corresponding to each isolate was selected based on the maximum score and/or number of hits they showed in the BLAST and RDP seqmatch search results. Organisms with an ambiguous description such as enrichment culture clones, uncultured or unclassified bacteria were not taken into consideration (Hossain et al., 2018). Taxonomic classification of the isolates based on the 16S rRNA genes was performed using RDP classifier program (Cole et al., 2014) with the confidence threshold setting at 80%.

### Construction of Phylogenetic Tree

Phylogenetic tree was constructed as previously described (Hossain et al., 2018). 16S rRNA gene sequences of type-strains (T) which are closely related to the enzyme-producing isolates were retrieved from EzBioCloud database (Yoon et al., 2017). The retrieved sequences and sequences of the isolated strains were aligned by MUSCLE or ClustalW algorithms in Geneious Prime 2019.2.3^1^. Phylogenetic tree of the aligned sequences was built using the maximum likelihood method (Aldrich, 1997) with Tamura-Nei distance algorithm in MEGA-X (Kumar et al., 2018) with 1000 bootstrap replicates. Trees were also calculated using two other algorithms such as parsimony and neighbor-joining with Jukes-Cantor correction in MEGA-X which produced same results.

## Results

### Distribution of Hydrolytic Enzyme Activities Among the Isolated Bacteria

**FIGURE 1:**
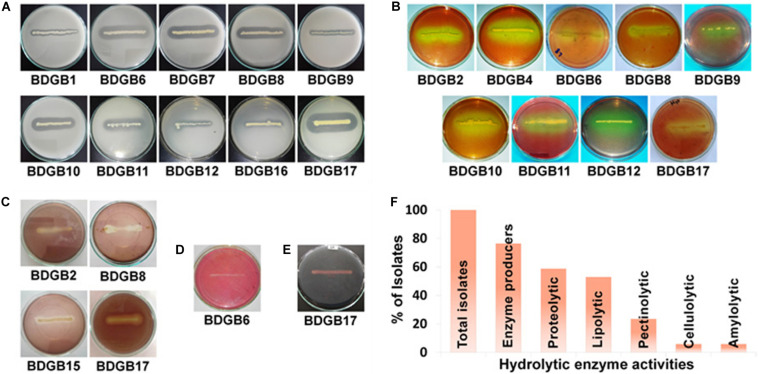
Production of extracellular hydrolytic enzymes by the gut-associated bacteria of Bombay duck. Plate assay showing **(A)** proteolytic, **(B)** lipolytic, **(C)** pectinolytic, **(D)** cellulolytic and **(E)** amylolytic enzyme activities of the isolated bacteria. The assay was performed by inoculation of the bacterial isolates on agar media containing suitable substrate specific for each enzyme activity. After incubation at 30°C for 48 h, media were treated with specific staining solutions to visualize formation of clear halos around bacterial colonies indicating production of the respective enzymes. Isolates that did not produce any halo zone, are not shown. **(F)** Distribution (%) of extracellular hydrolytic enzyme activities among the isolates.

The objective of the present work was to investigate the production of extracellular hydrolytic enzymes by bacteria associated with the GI tract of Bombay duck. Consequently, twenty bacterial strains, based on their divergence in the colony morphology, size and color, were isolated from the GI tract of this fish. 16S rRNA gene sequence analysis (described later), however, indicated that a few of these isolates were of same species and there were, in fact, seventeen different species among the isolates. The seventeen isolates, designated as BDGB1 to BDGB17, were screened for the presence of five different hydrolytic enzyme activities viz. proteolytic, lipolytic, pectinolytic, cellulolytic and amylolytic activities (Figures 1A–E). Of those, thirteen (∼77%) isolates were found expressing at least one of the five enzyme activities tested (Figure 1F). Distribution of the enzyme activities among the isolates is summarized in Table 1. Proteolytic activity was found to be the most common hydrolytic activity detected in a maximum of ten (∼59%) isolates, very closely followed by the lipolytic activity detected in nine (∼53%) of the isolates. Pectinolytic activity, however, was less frequently detected being produced by only four (∼24%) of the isolates whereas cellulolytic and amylolytic activity was very rare, found in one isolate each (Table 1 and Figure 1F). While none of the isolates expressed all the five hydrolytic enzymes, several of them produced two or more. A maximum of four hydrolytic enzymes were produced by only one isolate viz. BDGB17. Three different enzyme activities were detected in two of the isolates whereas two enzyme activities, which were generally protease and lipase, were detected in five isolates (Table 1). Five other isolates expressed only one hydrolytic enzyme which in most cases was either protease or lipase. The remaining four isolates, BDGB3, BDGB5, BDGB13, and BDGB14, did not show any of the enzyme activities tested.

**TABLE 1:** Hydrolytic enzyme profile of the gut-associated bacteria of Bombay duck.

**Isolated strains**	**Extracellular hydrolytic enzyme activities**				
	
	**Proteolytic**	**Lipolytic**	**Pectinolytic**	**Cellulolytic**	**Amylolytic**
BDGB1	+	−	−	−	−
BDGB2	−	+	+	−	−
BDGB4	−	+	−	−	−
BDGB6	+	+	−	+	−
BDGB7	+	−	−	−	−
BDGB8	+	+	+	−	−
BDGB9	+	+	−	−	−
BDGB10	+	+	−	−	−
BDGB11	+	+	−	−	−
BDGB12	+	+	−	−	−
BDGB15	−	−	+	−	−
BDGB16	+	−	−	−	−
BDGB17	+	+	+	−	+

*“+” and “−” indicate the presence and absence of the enzyme activities, respectively. BDGB3, BDGB5, BDGB13, and BDGB14 that did not produce any of the five hydrolytic enzymes, are not included.*

### Taxonomic and Hydrolytic Diversity of the Enzyme Producing Isolates

**FIGURE 2:**
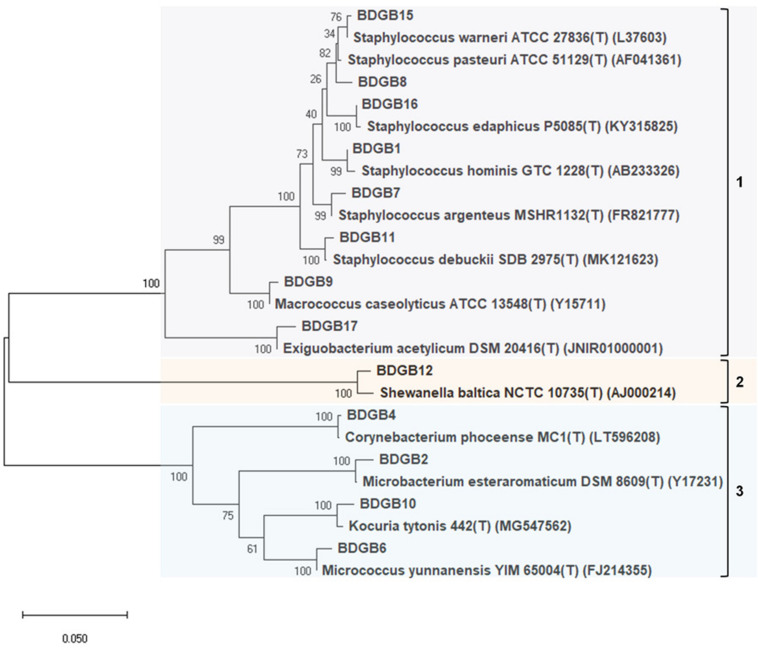
Phylogenetic tree constructed based on partial 16S rRNA gene sequences indicating evolutionary relationship of the enzyme-producing isolates among themselves and with their nearest type strains. Sequences were aligned with MUSCLE or ClustalW algorithms and a maximum-likelihood tree of the alignment was constructed using the Tamura-Nei model. Bootstrap values (%), indicated at branches, were calculated from 1000 replicates. Accession numbers of the type strains are given in parentheses after the strain names.

**FIGURE 3:**
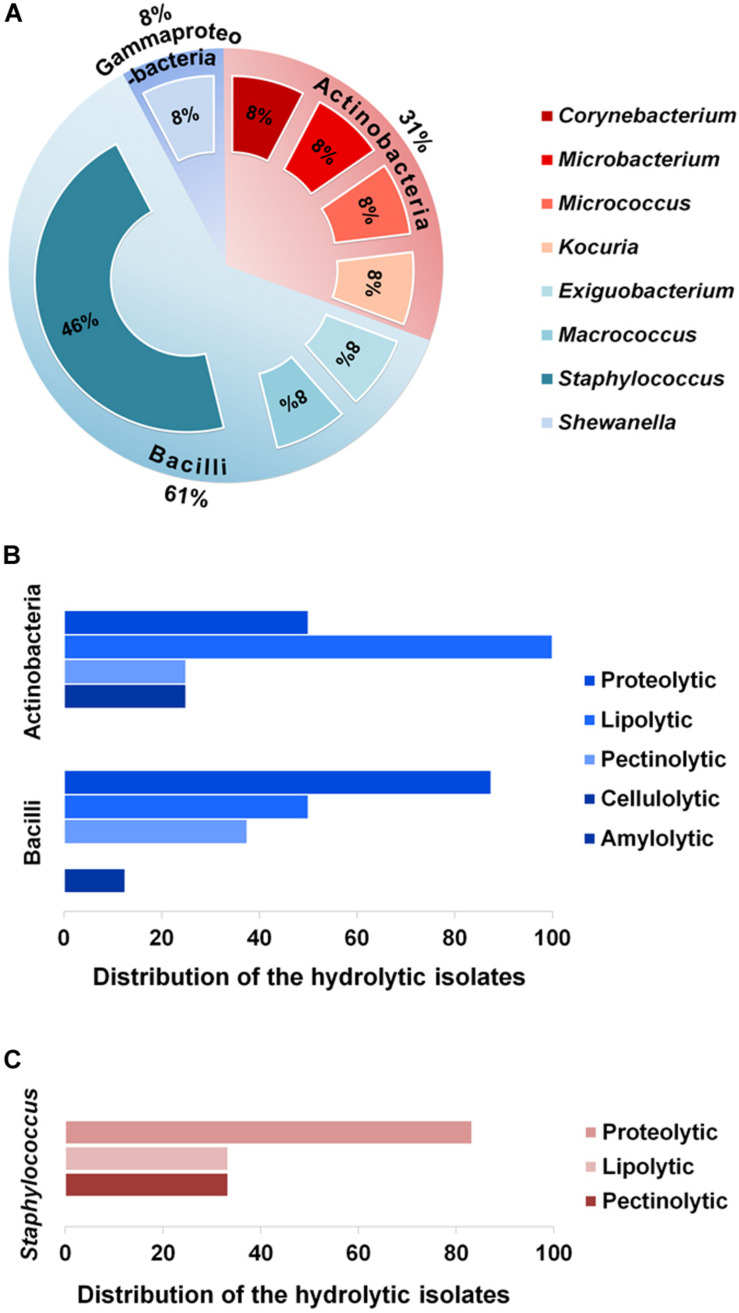
Proportions of the various phylotypes of the enzyme-producing isolates, and distribution of the isolates across these phylotypes. **(A)** Proportions (%) of the taxonomic classes and genera representing the thirteen enzyme-producing isolates. **(B)** Distribution (%) of the hydrolytic isolates across the two major classes, i.e., Actinobacteria and Bacilli. **(C)** Distribution (%) of proteolytic, lipolytic and pectinolytic isolates across the major genus, *Staphylococcus*.

To explore taxonomic diversity of the enzyme-producing isolates in the GI tract of Bombay duck, sequences of their 16S rRNA genes were analyzed. The sequences of the seventeen isolates were clustered into thirteen different operational taxonomic units (OTUs) based on >99% sequence identity. The sequence homology of the isolates and the sequence-based taxonomic identity were determined and presented in Table 2. The isolated strains showed more than 99% homology to the nearest neighboring sequences and at least 98% homology to the closest type strain sequences, supporting the taxonomic annotation to minimum genus level. The 16S rRNA gene-based phylogenetic tree illustrating evolutionary relationships among the enzyme-producing isolates, revealed that the thirteen isolates were distributed into three main clusters (Figure 2). Similarly, taxonomic classification of the isolates based on their 16S rRNA gene sequences also suggested that the isolates were grouped into three taxonomic classes including Actinobacteria, Bacilli and Gammaproteobacteria (Figure 3A) with Actinobacteria being the most diverse and Bacilli the largest. The four isolates of Actinobacteria were each affiliated to a different genus such as *Corynebacterium*, *Kocuria*, *Microbacterium*, and *Micrococcus*. On the other hand, Bacilli, accommodating a maximum of eight enzyme-producing isolates (61.5%), was rather less diverse, represented by only three different genera including *Staphylococcus* (six isolates), *Macrococcus* (one isolate) and *Exiguobacterium* (one isolate). The remaining phylotype, Gammaproteobacteria, included just one genus, i.e., *Shewanella* (one isolate). *Staphylococcus*, which was represented by ∼46% of the enzyme-producing isolates, was the most dominant among the eight genera (Figure 3A). Interestingly, most (∼67%) isolates belonging to *Staphylococcus* expressed only one of the five hydrolytic enzymes. In contrast, the rest of the genera were each consisted of a single isolate but most (∼86%) of them showed multi-enzyme capacity. The proteolytic and the pectinolytic strains, however, mostly fell in the *Staphylococcus* group (see Supplementary Figure S1). The 16S rRNA gene-based taxonomic affiliations also revealed that all of the identified isolates were Gram-positive bacteria with medium to high GC-content (50.8 to 57.2%), except only the *Shewanella* sp. which was Gram-negative.

**TABLE 2:** Taxonomic classification of the enzyme producing isolates based on sequence-homology of their 16S rRNA genes to the database sequences.

**Isolates**	**BLASTn**			**RDP**		**EzBioCloud taxonomy**		
	
	**Top Hit^a,b^**	**Query Cover (%)**	**Identity (%)**	**Classifier**	**SeqMatch score**	**Top Hit^a^ (Type strain)**	**Completeness (%)**	**Similarity (%)**
BDGB1	*Staphylococcus hominis* (PK2-4.1)	99	99.93	*Staphylococcus*	1.000	*Staphylococcus hominis* [GTC 1228(T)]	91.2	99.7
BDGB2	*Microbacterium esteraromaticum* (MM40)	99	99.34	*Microbacterium*	0.975	*Microbacterium esteraromaticum* [DSM 8609(T)]	94.3	99.05
BDGB4	*Corynebacterium sp.* (T2-10)	100	99.70	*Corynebacterium*	0.983	*Corynebacterium phoceense* [MC1(T)]	91.8	99.62
BDGB6	*Micrococcus sp.* (XHTSA12)	99	99.55	*Micrococcus*	1.000	*Micrococcus yunnanensis* [YIM 65004(T)]	92	99.32
BDGB7	*Staphylococcus aureus* (RM AST SA004)	100	99.44	*Staphylococcus*	1.000	*Staphylococcus argenteus* [MSHR1132(T)]	97.1	99.3
BDGB8	*Staphylococcus pasteuri* (JS7)	100	99.40	*Staphylococcus*	0.992	*Staphylococcus pasteuri* [ATCC 51129(T)]	90.4	99.4
BDGB9	*Macrococcus caseolyticus* (GCF1S3)	100	99.65	*Macrococcus*	0.983	*Macrococcus caseolyticus* [ATCC 13548(T)]	97	99.65
BDGB10	*Kocuria rhizophila* (8d–S5)	99	99.93	*Kocuria*	0.993	*Kocuria tytonis* [442(T)]	96.1	98.82
BDGB11	*Staphylococcus piscifermentans* (PCM 2409)	99	99.62	*Staphylococcus*	0.984	*Staphylococcus debuckii* [SDB 2975(T)]	90.4	99.55
BDGB12	*Shewanella baltica* (BA175)	100	99.79	*Shewanella*	1.000	*Shewanella baltica* [NCTC 10735(T)]	96.4	98.66
BDGB15	*Staphylococcus warneri* (20)	100	99.79	*Staphylococcus*	1.000	*Staphylococcus warneri* [ATCC 27836(T)]	96.9	99.37
BDGB16	*Staphylococcus saprophyticus* (1A)	100	99.62	*Staphylococcus*	0.994	*Staphylococcus edaphicus* [P5085(T)]	90.2	99.77
BDGB17	*Exiguobacterium acetylicum* (ESM25)	100	99.23	*Exiguobacterium*	1.000	*Exiguobacterium acetylicum* [DSM 20416(T)]	95.8	99.29

*^*a*^Strain names of the top hit species are given inside brackets. ^*b*^The *E*-values of all BLAST results were 0.0.*

On the other hand, analysis of enzymatic diversity in the two major taxonomic classes, Actinobacteria and Bacilli, suggested that the isolates of Bacilli were mostly proteolytic (87.5%), whereas half of the isolates of Actinobacteria were proteolytic but all of them expressed lipolytic activities (Figure 3B). The extent of hydrolytic activity at the genus level showed that more than 80% of the *Staphylococcus* sp. were proteolytic but none of the six *Staphylococcus* isolates was found to be cellulolytic or amylolytic (Figure 3C).

### Degree of Hydrolytic Capacity

To reveal which of the isolates produced higher amount of the enzymes, degree of their hydrolytic capacity for degradation of a given substrate were determined and presented as enzyme intensity (EI) in Table 3. On media containing gelatin as the substrate, BDGB11 showed the highest protease production (EI 5.22), followed by BDGB6 (EI 5.08). Lipase production, based on the depolymerization of Tween 80, was generally found higher; all strains showed an EI of at least 5.0 (Table 3). The maximum lipase production was detected in BDGB17 followed by BDGB4 (EI 8.89 and 7.45, respectively). Three other isolates, BDGB2, BDGB9 and BDGB11, also showed relatively high lipase production (EI ∼7.3). Similar to the lipase production, BDGB17 showed maximum pectinase production also (EI 6.67). Cellulolytic and amylolytic activities, which were detected in one isolate each viz. BDGB6 and BDGB17, respectively, were, however, produced in very small amounts (EI 1.83 and 2.06, respectively).

**TABLE 3:** Amount of extracellular hydrolytic enzymes produced by the bacterial isolates expressed as enzyme intensity (EI).

**Isolates**	**Enzyme Intensity (EI)**				
	
	**Protease**	**Lipase**	**Pectinase**	**Cellulase**	**Amylase**
BDGB1	2.67 ± 0.38				
BDGB2		7.22 ± 2.54	5.31 ± 0.93		
BDGB4		7.45 ± 1.26			
BDGB6	5.08 ± 0.14	5.42 ± 0.72		1.83 ± 0.29	
BDGB7	5.00 ± 0.25				
BDGB8	3.87 ± 0.11	6.70 ± 0.75	4.17 ± 0.14		
BDGB9	1.97 ± 0.05	7.30 ± 0.82			
BDGB10	4.89 ± 0.19	5.08 ± 0.14			
BDGB11	5.22 ± 0.19	7.22 ± 2.41			
BDGB12	4.83 ± 0.29	5.33 ± 0.58			
BDGB15			2.00 ± 0.43		
BDGB16	1.92 ± 0.14				
BDGB17	4.33 ± 0.08	8.89 ± 0.96	6.67 ± 0.33		2.06 ± 0.42

*Data represent the mean ± standard deviation of three independent experiments.*

Analysis of taxonomic affiliations of the high-produces, e.g., BDGB2, BDGB4, BDGB6, BDGB9, BDGB11, and BDGB17, revealed that they all came from different genera including *Microbacterium*, *Corynebacterium*, *Micrococcus*, *Macrococcus*, *Staphylococcus* and *Exiguobacterium*, respectively (Table 2). Three of these high-producers (BDGB2, BDGB11, and BDGB17) are particularly worthy of mention because they showed higher expressions of two different enzymes. For example, BDGB2 and BDGB17 both exhibited relatively high expression of lipase and pectinase enzymes, whereas BDGB11 produced higher amounts of protease and lipase enzymes (Table 3). It is also interesting to find that the lipolytic activity was expressed not only by a large number of the isolates; it was also produced in amounts higher than all the other hydrolases by most of these isolates (Table 3).

## Discussion

Majority (77%) of the bacteria isolated in this work produced at least one of the five hydrolytic enzymes (Figure 1). The most common of these enzymes were the ones related to the degradation of lipids and proteins. This enzymatic pattern appears to be strongly correlated to the eating behavior of Bombay duck. Being a carnivore, Bombay duck mainly feeds on small animals such as zooplanktons (Zhang and Jin, 2014) suggesting that proteins and lipids comprise major fractions of its diet. With plenty of the dietary substrates available for digestion, predominance of the proteolytic (59%) and lipolytic (53%) strains in the GI tract of Bombay duck is normal. This also explains why the cellulolytic and amylolytic activities were found in very small number of the bacteria; lack of the plant-based macromolecules such as cellulose, starch etc. in the diet of Bombay duck implies that the bacteria which rely on their extracellular cellulolytic and/or amylolytic enzymes for nutrition, might not get sufficient nutrients due to the lack of suitable substrates in the host diet. Therefore, the cellulolytic or amylolytic microbes gaining entry into the GI tract, might not want to establish themselves there. Previously, Bairagi et al. (2002) also failed to detect cellulolytic activity in the gut bacteria of two carnivorous fishes, murrel and walking catfish. Moreover, each of the isolates having cellulolytic (BDGB6) or amylolytic (BDGB17) activity in this study, also showed both of the proteolytic and lipolytic activities (Table 1), suggesting that these strains are not exclusively or inevitably dependent on their cellulolytic or amylolytic activity for nutrition. This is also consistent with the finding that the production of the cellulase and amylase enzymes of these isolates was much lower than that of the lipase or protease enzymes (Table 3). Surprisingly, however, pectinolytic activity that degrades pectin, a plant-based biopolymer, was detected in rather higher number (24%) of the isolates. While three of these isolates (BDGB2, BDGB8, and BDGB17) also showed proteolytic and/or lipolytic activities, the remaining one (BDGB15) didn’t show any additional hydrolytic activity (Table 1). It might be possible that one or more of these pectinolytic strains were transient or occasional visitors (allochthonous) to the GI tract. Reports describing the pectinolytic strains in fish GI tract are, in fact, very scarce. Before the present work, only two groups (Mountfort et al., 1993; Sasmal and Ray, 2015) described the presence of pectinolytic activity in bacteria isolated from the gut of mullet and Tilapia. In general, the percentage of bacteria showing extracellular lipolytic and proteolytic activities in this work is pretty similar to that reported in other studies (Gatesoupe et al., 1997; Sumathi et al., 2011; Ariole et al., 2014; Tiwari et al., 2015). However, findings regarding the percentage of cellulolytic and amylolytic strains are quite different from those in a number of previous reports which described higher proportions of these isolates even in carnivorous fishes (Das and Tripathi, 1991; Sugita et al., 1997; Saha and Ray, 1998; Bairagi et al., 2002; Saha et al., 2006; Mondal et al., 2008, 2010; Ray et al., 2010; Das et al., 2014; Banerjee et al., 2016). In any case, the profile of hydrolytic activities of the isolates revealed in this study, clearly indicates that the gut microbiota of Bombay duck play a very important function in the digestion of the foods it consumes, thus providing essential nutrients to the host. Moreover, the proteolytic populations, in addition to their function in the digestive process, are also known to contribute via their antagonistic and lytic activities against other bacteria and therefore considered very useful as probiotics in aquaculture.

All the isolates identified in this work had 16S rRNA gene sequences highly similar to previously reported GenBank sequences showing over 99% homology in BLAST’s similarity search (Table 2). For each isolate it was found that, one or more of its closest relatives that existed within the topmost bacteria in BLAST’s search-result, were also collected from marine environment. For example, *S. hominis* strain P12-B3-1 (ACN: MK318620), *M. esteraromaticum* strain BA1109 (KC430861), *C. phocae* strain M408/89/1 (CP009249), *M. yunnanensis* strain P1-A7 (MK318591), *S. aureus* strain AM (MG230264), *Staphylococcus* sp. strain SA02-L01c (MN043814), *M. caseolyticus* strain GCF1S3 (MG744632), *K. rhizophila* strain FEB3-08 (MG780345), *Staphylococcus* sp. strain S8 (MK954148), *S. baltica* strain BA175 (CP002767), *S. saprophyticus* strain P0081Karwar, uncultured *Exiguobacterium* sp. clone CD25 (KF760547) etc. were all obtained from various marine sources such as sea sediment, sea water, intestinal content of marine fish etc. This finding is consistent with the origin of the BDGB isolates as marine-derived organisms.

Some of the species identified in the gut of Bombay duck, have been previously shown to exert beneficial effects in other fish. For example, biosurfactant isolated from *S. hominis* significantly enhanced specific and non-specific immunity and disease resistance in finfish (Rajeswari et al., 2016). In a study on the probiotic characterization of *S. baltica*, a significant increase in the growth of Senegalese sole was found when the fish was fed with a diet supplemented with the bacterial strain (Díaz-Rosales et al., 2009). Besides, an increased immune response as well as resistance against *Photobacterium damselae* was observed. *E. acetylicum*, another sp. identified in the present study, exhibited excellent probiotic qualities and is therefore considered as a strong candidate for probiotic applications in aquaculture (Jinendiran et al., 2019a, b). Jinendiran et al. demonstrated that dietary supplementation of *E. acetylicum* resulted in a significant enhancement of the growth, hematological profile and cellular immune responses including respiratory burst, phagocytic activities, total immunoglobulin levels and antimicrobial enzymes in goldfish (Jinendiran et al., 2019b). Moreover, both goldfish and brine shrimp when fed with *E. acetylicum* showed a significantly higher survival rate against *Aeromonas hydrophila* (Jinendiran et al., 2019a, b). The *E. acetylicum* sp. has also been shown to be involved in the innate immune response in Zebrafish (Murdoch and Rawls, 2019).

Considering hydrolytic capacity, the *Exiguobacterium* sp. (BDGB17) appeared to be the most promising strain of all the bacteria isolated in the present study. It expressed maximum number of the hydrolytic enzymes and also showed highest production of two of those hydrolases. *Exiguobacterium* is comparatively a newly-described genus; yet several of its species have already gained much attention for their unique properties that are useful in the biotechnology, bioremediation, agriculture and industrial processes (Kasana and Pandey, 2018). In accordance with the results found in this study, other reports also described production of hydrolytic enzymes by *Exiguobacterium* sp. such as protease, lipase, pectinase and amylase, in addition to other hydrolases (Kasana and Pandey, 2018) not investigated in this research. The strain described by Vijayalaxmi et al. was able to produce cellulase (Vijayalaxmi et al., 2013), though BDGB17 of the present study showed negative results in the cellulase plate assay under the culture condition used. Spp. of *Exiguobacterium* were isolated from a wide range of environments including few marine sources (Kasana and Pandey, 2018) but before this work, only one study documented the presence of this sp. in the gastrointestinal tract of vertebrates (Jammal et al., 2017). Its identification in the gut of *Liza aurata* in that study was, however, not conclusive at the species level showing 97% similarity of the 16S rRNA gene sequence with the closest match. Two other isolates of the present work, BDGB6 and BDGB8 which produced the second-highest number of the hydrolases, belonged to *Micrococcus* and *Staphylococcus* groups, respectively. Strains belonging to these two groups were, however, reported to be isolated from fish digestive tract and are also known to produce a number of different extracellular hydrolytic enzymes (Ray et al., 2012).

The bacteria of this work were found moderately diverse in their taxonomic groups. In fact, bacterial diversity in the fish gut is generally lower in carnivores, and progressively increases in omnivores and herbivores (Liu et al., 2016; Butt and Volkoff, 2019). Moreover, bacteria identified in the present work were obtained from two fish; inclusion of more fish might help in a better rationalization of the microbial diversity in fish gut. The identified species represent three different bacterial phyla: Firmicutes, Actinobacbacteria and Gammaproteobacteria. These three phyla have been reported to predominate in the gut microbiota of many other fishes such as sea bream, grass carp, rainbow trout, coho salmon, zebrafish etc. (Rawls et al., 2004; Romero and Navarrete, 2006; Navarrete et al., 2009, 2012; Wu et al., 2012; Estruch et al., 2015). Moreover, similar to the findings of the present study, members of the three phyla were also found to make an important contribution to the digestive process of the host by providing a variety of hydrolytic enzymes (Hamid et al., 1979; Gatesoupe et al., 1997; Bairagi et al., 2002; Navarrete et al., 2012; Ray et al., 2012; Egerton et al., 2018; Butt and Volkoff, 2019). No sp. from Bacteroidetes was, however, identified in the present work though members from this phylum are usually common being reported previously in seabass, *Kyphosus cinerascens* etc. (Carda-Diéguez et al., 2014). Spp. of the less common phyla such as Fusobacteria, Flavobacteria etc. (Llewellyn et al., 2014; Wang et al., 2018) were also absent among the hydrolytic bacteria isolated in the present study. In fact, the bacterial diversity in fish gut depends on a variety of factors (Egerton et al., 2018; Talwar et al., 2018). One of the most important of these factors is the type of the host diet which in turn depends on the feeding behavior of the fish and both have been found to directly influence the microbiota structure of GI tract (Ray et al., 2012; Egerton et al., 2018; Butt and Volkoff, 2019). Connection between the host diet and gut microbial composition is clearly evident from the findings of the present study also, as already discussed. Another important factor, in addition to the host diet and trophic level, is the environmental condition of the habitat such as the water temperature (Egerton et al., 2018). Previously Huyben et al. showed that raising water temperature from 11 to 18°C resulted in an increase of both bacterial abundance and diversity in the gut microbiota of rainbow trout, while the optimal growth temperature of the fish is as high as 17°C (Huyben et al., 2018). Waché et al. (2006) also reported a change in the gut bacterial load in rainbow trout resulting from changes in water temperatures. Other reports suggested, however, that increase of water temperature above 17°C can induce stress, reduce growth and may disrupt microbial communities in the gut of this fish (Huyben et al., 2018). The habitat of Bombay duck includes the tropical waters of Indo-Pacific including Indian Ocean and the Bay of Bengal (Zhang et al., 2017). Waters in this region maintain temperatures of >28°C at the sea-surface the whole year round (De Deckker, 2016). Besides, those waters maintain such temperatures down to approximately 200 m in most regions, an environment that appears to be preferred by Bombay duck (Sundara Raj, 1954; Bapat, 1970; De Deckker, 2016). Hence, bacteria in the present study were isolated and grown at 30°C to match the temperature of their natural environment. Some of these bacteria were also isolated from the gut of other marine and fresh water fishes. For example, in an analysis of the GI microbiota of Mediterranean fish, Jammal et al. (2017) reported identification of *S. baltica* in seven out of the fifteen fish species they’ve examined including *Sargocentron rubrum*, *Dentex macrophthalmus*, *Dicologlossa cuneata*, *Oblada melanura*, *Pempheris rhomboidea*, *Sardinella maderensis*, and *Lithognathus mormyrus*. In fact, the *Shewanella* sp. appears to be a bacterium frequently found in the GI microbial community of fishes. In addition to the above seven fish species, this bacterium was also identified in the intestine of lean lake trout, pufferfish and whitefish (*Coregonus clupeaformis*) (Dailey et al., 2016; Castillo et al., 2018; Li et al., 2020). Alongside *S. baltica*, Jammal et al. identified two other spp. of the present work: *S. hominis* and *K. rhizophila* that were isolated from *S. rubrum* and *S. rivulatus*, respectively. In fact, the *Staphylococcus* spp. seem pretty common in the fish intestine. In addition to the *S. hominis* sp. described above, three more *Staphylococcus* spp. of the present investigation were identified in the gut of other fish species. *S. warneri* and *S. pasteuri*, for example, were reported in the intestine of Atlantic salmon in several individual research (Bakke-McKellep et al., 2007; Askarian et al., 2011; Abid et al., 2013); the latter sp. was also identified in the intestine of gibel carp (*Carassius auratus*) as well as in the fresh water fish Nile tilapia (He et al., 2011; Wu et al., 2020). Fish in those studies were, however, fed with specific diets. Another *Staphylococcus* species of this study, *S. saprophyticus*, was reported in the intestine of a variety of fish species including flatfish, butterfly peacock bass, Malaysian mahseer, orange-spotted grouper, Mozambique tilapia and Nile tilapia (Silva and Widanapathirana, 1984; Sun et al., 2011; Sivakumar et al., 2015; Nosi et al., 2018; Wanka et al., 2018). Therefore, the *S. saprophyticus* isolate together with *S. baltica* appear to be very common members of fish GI microbiota, suggesting that they both are autochthonous species.

In summary, this study showed that the GI tract of Bombay duck harbors diverse bacterial populations producing various extracellular hydrolytic enzymes including proteases, lipases and pectinases in particular. The enzyme producing capacity of the isolates suggests that the gut bacterial flora serves as an important source of digestive enzymes for the host and plays important roles in the host’s digestive metabolism and nutrition. The results also highlight the importance of fish GI microbiota for bioprospection of industrially useful enzymes. The isolates that showed high activities in this study merit further investigations to understand their actual capacity to produce the hydrolytic enzymes which could lead to their important industrial applications.

## Data Availability Statement

The datasets presented in this study can be found in NCBI Data Bank. The accession number(s) can be found in the article/Material and Methods.

## Author Contributions

TH conceived and supervised the project. TH and SC designed the experiments. SC, TH, HM, MC, FA, NR, and SD conducted the experiments. TH analyzed the results and prepared and wrote the manuscript. All authors reviewed and approved the manuscript.

## Conflict of Interest

The authors declare that the research was conducted in the absence of any commercial or financial relationships that could be construed as a potential conflict of interest.

## Footnotes

1 https://www.geneious.com
