# Supplementary material for: Hydrolytic Exoenzymes Produced by Bacteria Isolated and Identified From the Gastrointestinal Tract of Bombay Duck
Source: Front Microbiol. 2020 Aug 26;11:2097. doi: 10.3389/fmicb.2020.02097 (PMC7479992; doi:10.3389/fmicb.2020.02097)
Supplement: Supplementary file 1 [file Image_1.pdf]

Supplementary Material

1 Supplementary Figures

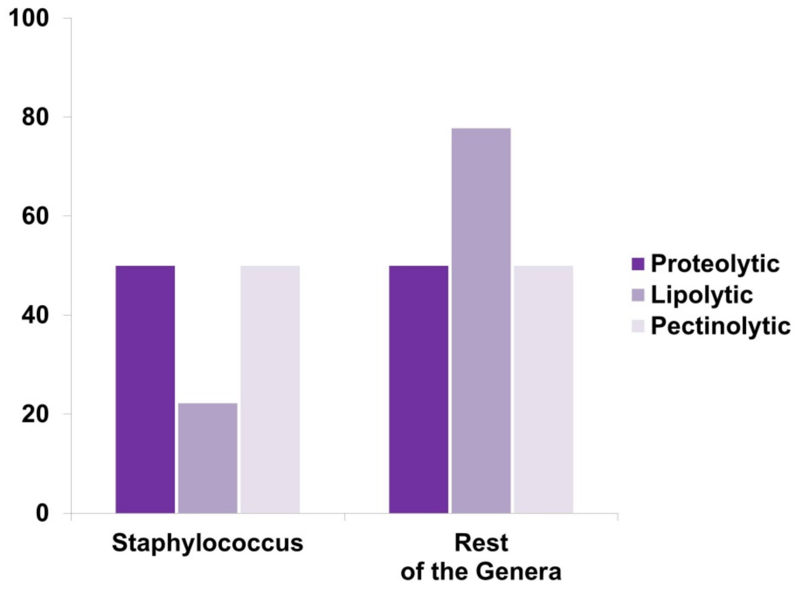

Supplementary Figure 1. Proportions (%) of the proteolytic, lipolytic and pectinolytic isolates among the genera.
